# Different clinical patterns of IgG4-RD patients with and without eosinophilia

**DOI:** 10.1038/s41598-019-52847-6

**Published:** 2019-11-11

**Authors:** Xia Zhang, Panpan Zhang, Jieqiong Li, Yujie He, Yunyun Fei, Linyi Peng, Qun Shi, Wen Zhang, Yan Zhao

**Affiliations:** 1 Department of Rheumatology, Peking Union Medical College Hospital, Chinese Academy of Medical Science & Peking Union Medical College, Key Laboratory of Rheumatology and Clinical Immunology, Ministry of Education, Beijing, China; 2 Department of Rheumatology, First Affiliated Hospital of Zhengzhou University, Zhengzhou, China

**Keywords:** Rheumatic diseases, Rheumatic diseases, Connective tissue diseases

## Abstract

It has been reported that patients with IgG4-related disease (IgG4-RD) showed an elevated incidence of eosinophilia. We aim to explore the clinical patterns of IgG4-RD patients with and without eosinophilia. Four hundred and twenty-five IgG4-RD patients referred to Peking Union Medical College Hospital were enrolled. Blood eosinophil count higher than 0.5 × 10^9^/L was defined as eosinophilia. Clinical features of all the participants were collected and analyzed statistically. Eighty-seven patients (20%) with eosinophilia were found. As compared to those with a normal range of blood eosinophil count, male predominance, longer disease duration, increased prevalence of dacryoadenitis, sialadenitis, lymphadenopathy, and skin rash, higher IgG4-RD responder index, more organ involvement and higher levels of serum IgG4 (17.0 g/L vs 6.5 g/L, *P* < 0.001) was found in patients with eosinophilia. There was no significant difference in the incidence of allergic disease between the two groups. Peripheral eosinophil counts were positively correlated with disease duration, the number of involved organs, IgG4-RD responder index, and serum IgG4. Higher recurrence rate during follow-up period was found in patients with eosinophilia [28.6% (20/70) vs 17.1% (42/245), *P* = 0.034]. IgG4-RD patients with eosinophilia exhibited different clinical patterns from patients without. Eosinophilia appeared independent of allergies in IgG4-RD.

## Introduction

Eosinophils are terminally differentiated cells of the myeloid lineage implicated in the pathogenesis of numerous inflammatory processes^1^. In response to a variety of stimuli, mature peripheral blood eosinophils are recruited into the tissue, where they modulate immune responses through multiple mechanisms. Eosinophils secrete a series of cytokines capable of promoting T cell expansion, and T helper type 1 (Th1)/2 (Th2) polarization^2^. Eosinophilia is defined as an elevation of eosinophils in the bloodstream. Many diseases are associated with eosinophilia, including parasitic diseases, allergy, autoimmune diseases, malignancy, primary hypereosinophilic syndrome^3^.

Immunoglobulin G4-related disease (IgG4-RD) is an increasingly recognized chronic fibroinflammatory disorder with multiple organ involvement, including salivary glands, lacrimal glands, pancreas, retroperitoneum, kidneys, lymph nodes, lungs, and liver among others. IgG4-RD is pathologically characterized by IgG4-positive lymphoplasmacytic infiltration, storiform fibrosis and obliterative phlebitis^4^. The pathogenesis of IgG4-RD remains poorly understood.

Recently, eosinophilia had been reported to be associated with IgG4-RD to varying degrees (11–38%)^5–11^. This eosinophilia appeared inherent to the IgG4-RD rather than atopic disease^10,11^. Some patients with high eosinophils as the first manifestation were finally confirmed to be IgG4-RD^12–14^. Eosinophils also infiltrate in the involved tissues, which is generally mild to moderate but can be remarkable in some cases^15^. Eosinophilic angiocentric fibrosis was recently described as a form of IgG4-related systemic disease^16^. In 2010, Sah *et al*. reported a similar clinical profile in type 1 autoimmune pancreatitis patients with and without peripheral eosinophilia^11^. In 2014, Della-Torre *et al*. reported that there was a positive correlation between eosinophil count and serum IgG4^10^. In 2017, Culver *et al*. revealed that eosinophil count was positively correlated with both serum IgE and serum IgG4. However, there was no statistical difference in serum IgG4 levels between IgG4-RD patients with and without eosinophilia^7^.

In this study, we investigated the prevalence of eosinophilia in IgG4-RD patients in the largest prospective IgG4-RD cohort in China, and report here for the first time that patients with eosinophilia presented with significantly different clinical patterns in comparison to those with normal peripheral eosinophil count.

## Results

### Patients with eosinophilia showed male predominance and longer disease duration

Among 425 patients in this cohort, the median blood eosinophil count was 210 cells per μL (IQR 100–420; Table 1). Eighty-seven patients (20%) showed peripheral blood eosinophil count higher than 500 per μL (0.5 × 10^9^ per L), 134 patients (32%) showed peripheral blood eosinophil ratio more than 5%. The eosinophil count higher than 1500 cells per μL were recorded in 13 (3%) patients.

**Table 1:** Baseline demographic and clinical characteristics of patients with IgG4-RD.

	Total (n = 425)	Patients without eosinophilia (n = 338)	Patients with eosinophilia (n = 87)	P value
Peripheral blood eosinophil count (cells per μL)	210 (100–420)	170 (80–260)	770 (610–1100)	<0.001
Age (years)^#^	54 ± 13	54 ± 13	53 ± 15	0.442
Male (%)	60.4%	57.1%	73.3%	0.007
Disease duration (months)^*^	12(4–36)	10(4–36)	12(6–48)	0.034
Allergy history	49.2%	47.6%	55.2%	0.145
IgG4-RD RI^*^	12 (7–16)	11 (7–15)	13 (10–17)	<0.001
Numbers of organs involved	3.31 ± 1.71	3.17 ± 1.63	3.86 ± 1.89	0.003
HBG (g/L)^#^	134.2 ± 18.3	134.2 ± 18.1	134.3 ± 19.2	0.687
PLT (×109/L)^*^	231 (195–280)	231 (191–281)	237 (206–278)	0.254
WBC (×10^9^/L)^*^	6.7 (5.6–8.0)	6.5 (5.5–7.7)	7.32 (5.90–9.03)	<0.001
ESR (mm/h)^*^	18 (8–51)	17 (8–44)	34 (12–76)	0.002
CRP (mg/L)^*^	2.1 (0.8–7.6)	2.3 (0.7–7.6)	2.1 (1.3–7.4)	0.342
IgG (g/L)^*^	18.7 (14.6–24.7)	18.1 (14.2–23.3)	24.1 (16.4–32.6)	<0.001
IgA (g/L)^*^	2.06 (1.39–2.75)	2.14 (1.54–2.85)	1.54 (0.96–2.13)	<0.001
IgM (g/L)^*^	0.77 (0.55–1.22)	0.81 (0.57–1.24)	0.70 (0.48–1.05)	0.094
IgG1 (g/L)^*^	9.14 (7.45–11.00)	9.06 (7.30–10.60)	9.68 (7.65–13.10)	0.009
IgG2 (g/L)^*^	5.73 (4.42–7.58)	5.88 (4.56–7.64)	5.27 (3.75–7.18)	0.022
IgG3 (g/L)^*^	0.45 (0.24–0.86)	0.42 (0.23–0.81)	0.55 (0.31–0.98)	0.012
IgG4 (g/L)^*^	7.72 (3.18–17.50)	6.50 (2.74–14.38)	17.00 (5.60–31.55)	<0.001
IgG4/IgG^*^	0.34 (0.17–0.53)	0.31 (0.16–0.48)	0.49 (0.25–0.61)	<0.001
IgE (KU/L)^*^	347 (126–752)	302 (119–662)	480 (157–1164)	0.010
C3 (g/L)^*^	0.94 (0.75–1.11)	0.96 (0.79–1.11)	0.79 (0.58–1.15)	0.088
C4 (g/L)^*^	0.17 (0.12–0.24)	0.18 (0.13–0.24)	0.12 (0.05–0.19)	<0.001

HGB, hemoglobin; PLT, platelet cell; WBC, white blood cell; GC mono, glucocorticoids monotherapy; GC & IM, glucocorticoids combined immunosuppressants therapy; IM mono, immunosuppressants monotherapy; TCM, traditional Chinese medicine; *Values are median (IQR); ^#^Values are Mean ± Standard Deviation.

Patients with eosinophilia were more likely to be male (73.3% vs. 57.1%, *P* = 0.007) and with longer disease duration [12(6–48) vs. 10(4–36), *P* = 0.034] (Table 1).

### Patients with eosinophilia exhibited more organ involvement and higher serum IgG4 level

**Figure 1:**
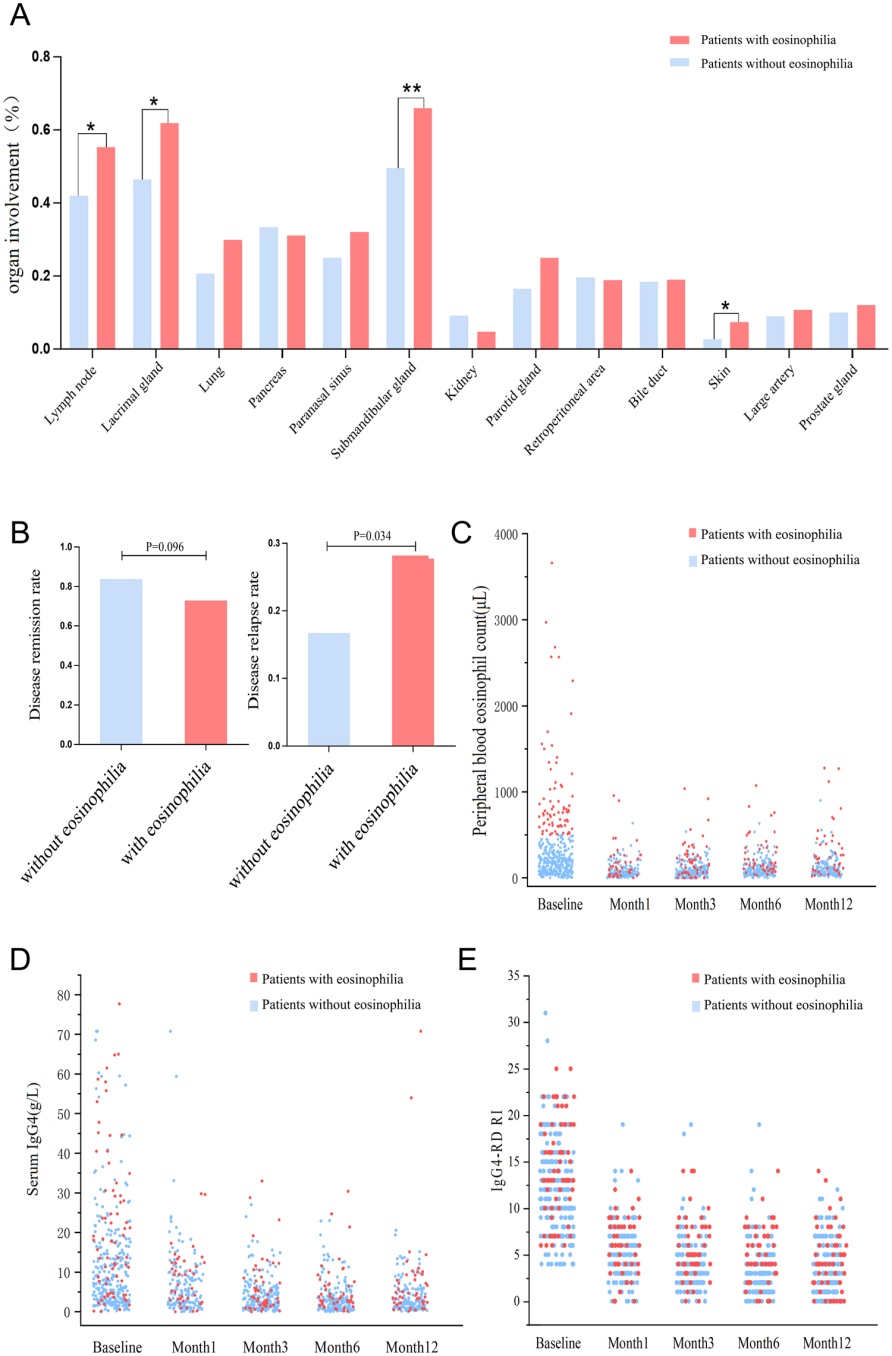
Organs involvement and outcome of patients with and without eosinophilia. (**A**) Organ involvement of the two groups of patients. (**B**) Disease remission rate and recurrence rate. (**C–E**) Changes of peripheral blood eosinophil count, serum IgG4, IgG4-RI of the two groups of patients during follow-up. IgG4-RD RI, IgG4-RD responder index. *P < 0.05; **P < 0.01.

Patients with eosinophilia had higher incidence of dacryoadenitis (61.9% vs 16.4%, *P* = 0.014), submandibular sialadenitis (65.9% vs 49.5%, *P* = 0.007), lymphadenopathy (55.3% vs 42.0%, *P* = 0.037) and skin rash (7.4% vs 2.7%, *P* = 0.034) (Fig. 1A), as well as higher IgG4-RD RI and more organ involvement (*P* < 0.05, Table 1). Interestingly, there was no significant difference in the incidence of allergic diseases between patients with and without eosinophilia (Table 1). In the laboratory inspection, patients with eosinophilia showed higher peripheral WBC, ESR, serum IgG, IgG1, IgG3, IgG4, IgE, and IgG4/IgG ratio, while lower serum IgA, IgG2 and C4 level (*P* < 0.05, Table 1).

### Peripheral eosinophil counts correlated to disease burden in IgG4-RD patients

Correlation analysis revealed that peripheral eosinophil counts were positively correlated with disease duration, numbers of organs involved, and IgG4-RD RI (Table 2). Besides, blood eosinophil counts were also positively related to peripheral WBC, ESR, IgG, IgG1, IgG3, IgG4, IgE, and IgG4/IgG ratio, whereas negatively correlated to IgA, IgM, IgG2 and C4, as showed in Table 2. All these indicate that peripheral eosinophil counts were associated with disease burden in IgG4-RD patients.

**Table 2:** Correlations between peripheral blood eosinophil count and clinical indicators.

Parameters	Peripheral blood eosinophil count	
	Spearman r	P value
Age (years)	0.005	0.922
Disease duration (months)	0.140	0.006
Numbers of organs involved	0.153	0.002
IgG4-RD RI	0.169	0.001
WBC (×10^9^/L)	0.190	<0.001
ESR (mm/h)	0.104	0.040
CRP (mg/L)	0.012	0.827
IgG (g/L)	0.240	<0.001
IgA (g/L)	−0.244	<0.001
IgM (g/L)	−0.128	0.010
IgG1 (g/L)	0.102	0.042
IgG2 (g/L)	−0.108	0.030
IgG3 (g/L)	0.115	0.021
IgG4 (g/L)	0.335	<0.001
IgG4/IgG	0.320	<0.001
IgE (KU/L)	0.179	0.002
C3 (g/L)	−0.106	0.094
C4 (g/L)	−0.200	0.002

### Patients with eosinophilia were more liable to undergo disease relapse

In the overall 425 patients, 46 patients (10.8%) were untreated and under observation, 110 patients (25.9%) received glucocorticoids monotherapy (GC mono), 60 patients (14.1%) received immunosuppressants monotherapy (IM mono), 208 patients (48.9%) received combination therapy with GC and IM, 1 patient (0.2%) chose traditional Chinese medicine treatment. Treatment options displayed no significant difference between the two groups.

Remission rates were evaluated six months after initiating remission induction treatment with GC or GC combined IM. It seemed that patients with eosinophilia tend to have a lower remission rate, but the difference was not statistically significant [83.8% (160/191) vs. 72.9% (35/48), *P* = 0.096] (Fig. 1B). IgG4-RD RI dropped by half or more at the 6^th^- month follow-up in the two groups were 92.2% (202/219) and 89.7% (52/58), respectively (*P* = 0.526).

Patients were followed up for 25 ± 15 months. The disease relapse rate was calculated in 315 patients who were followed up for three months or longer. The final results showed that the total recurrence rate was 19.7% (62/315), and patients with eosinophilia showed a higher relapse rate than patients without eosinophilia [28.6% (20/70) vs. 17.1% (42/245), *P* = 0.034] (Fig. 1A). The mean time of recurrence was around 12th (7–24) month, no significant difference was found between two groups [11 (6–24) month vs. 12 (7–27) month, *P* = 0.509]. Patients with hypereosinophilia (>1500 cells per μL) displayed a trend of higher recurrence rate than patients with blood eosinophil count range from 500 to 1500 cells per μL, but the difference was not significant [33.3% (4/12) vs. 27.6% (16/58), *P* = 0.768]. A lower proportion of patients with eosinophilia had a blood IgG4 level reaching the normal range at the 3rd, 6th, 12th-month follow-up (Table 3).

**Table 3:** Different levels of serum IgG4 during follow-up.

	Patients without eosinophilia	Patients with eosinophilia	P value
**Serum IgG4 at baseline**			
Normal (<1.35 g/L)	4.7% (16/338)	3.4% (3/87)	0.625
**Serum IgG4 at 1** ^**st**^ **month follow-up**			
Normal (<1.35 g/L)	17.7% (29/164)	13.5% (5/37)	0.430
Decrease by 50% or more*	47% (77/164)	54.1% (20/37)	0.626
**Serum IgG4 at 3** ^**rd**^ **month follow-up**			
Normal (<1.35 g/L)	34.7% (77/222)	15.8% (9/57)	0.003
Decrease by 50% or more*	72.5% (161/222)	80.7% (46/57)	0.317
**Serum IgG4 at 6** ^**th**^ **month follow-up**			
Normal (<1.35 g/L)	40.2% (78/194)	14.6% (7/48)	0.001
Decrease by 50% or more*	70.1% (136/194)	81.3% (39/48)	0.122
**Serum IgG4 at 12** ^**th**^ **month follow-up**			
Normal (<1.35 g/L)	37.8% (65/172)	19.1% (9/47)	0.011
Decrease by 50% or more*	73.8% (127/172)	70.2% (33/47)	0.620

*As compared to serum IgG4 levels at baseline.

**Figure 2:**
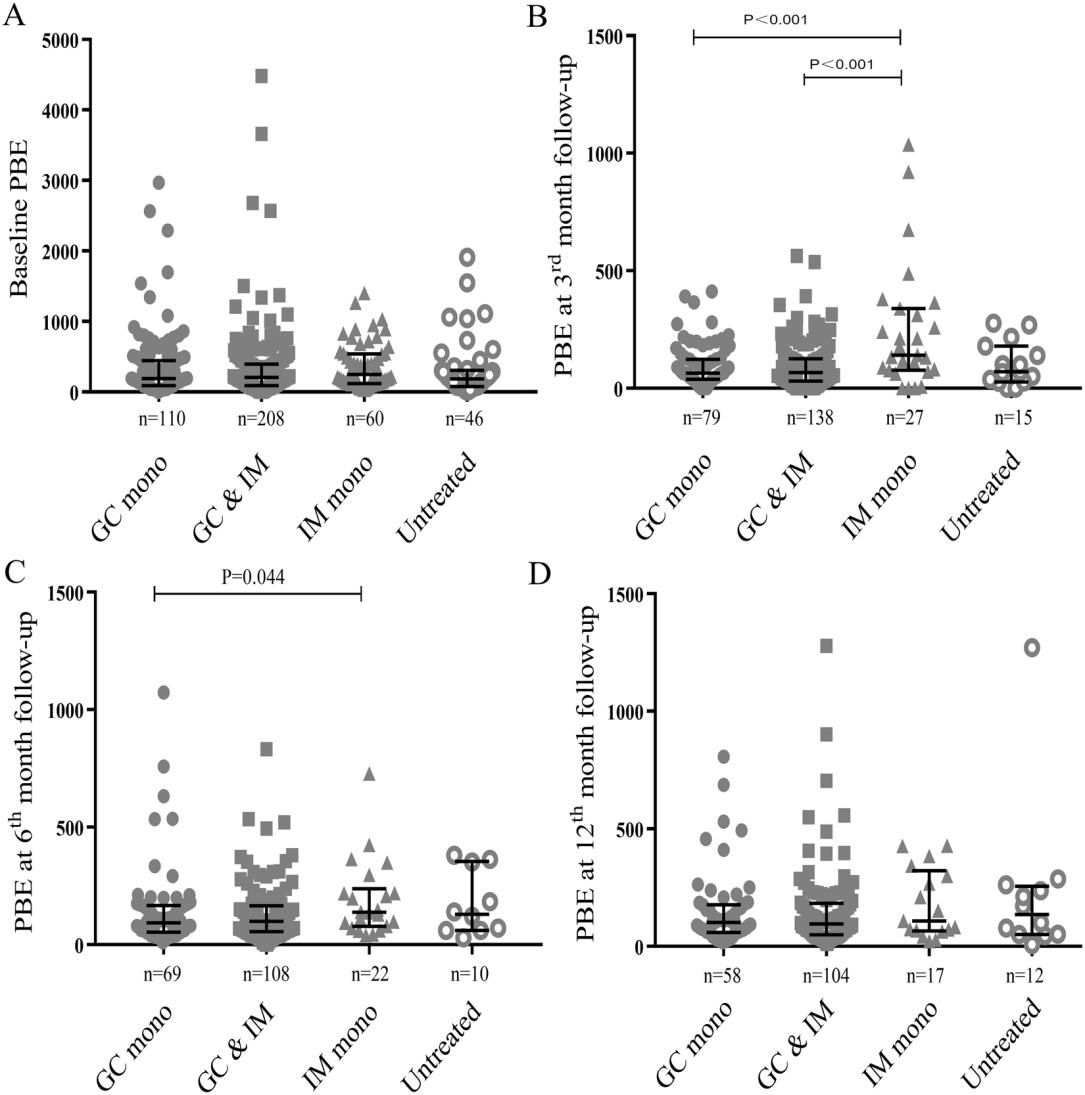
Changes in peripheral blood eosinophil (PBE) counts during follow-up, grouped according to treatment options. (**A–D**) display peripheral blood eosinophil counts at baseline, the third month follow-up, the sixth-month follow-up, and the 12th month follow-up, respectively. Bars represent median and interquartile ranges. GC mono, glucocorticoids monotherapy; GC & IM, combination therapy with glucocorticoids and immunosuppressants; IM mono, immunosuppressants monotherapy.

During the follow-up period, patients with eosinophilia improved significantly, and the peripheral blood eosinophils fell to normal range (<500/μL) in 92.6% (50/54), 80.9% (38/47) and 83% (35/42) at the 3rd, 6th, 12th-month follow-up, respectively. While three patients without eosinophilia at baseline assessment developed eosinophilia during follow-up, and these patients showed no signs of disease relapse (Fig. 1C). The trends of serum IgG4 and IgG4-RD RI levels during follow-up were shown in Fig. 1D,E. Univariable logistic regression analysis revealed that eosinophilia at baseline and IgA < 0.7 g/L (lower limit of the normal range) were two risk factors for sustained eosinophilia at the 12th-month follow-up (Table 4). Serum IgG4 level at 12 months was positively correlated with blood eosinophil count at 12 months (r = 0.314, *P* < 0.001). Comparing patients with different treatment choices, we found that glucocorticoid-based therapy seemed to be more conducive to peripheral blood eosinophil counts declination (Fig. 2).

**Table 4:** Univariable logistic regression analysis of risk factors for eosinophilia at 12^th^ month follow-up.

	Eosinophilia at 12^th^ month follow-up	
	OR (95% CI)	P value
Female sex	0.18 (0.02–1.43)	0.105
Allergy history	0.86 (0.24–3.06)	0.812
Involved organ number	1.25 (0.75–2.10)	0.394
Eosinophilia at baseline	9.80 (2.41–39.81)	0.001
Elevated ESR	1.43 (0.39–5.25)	0.590
Elevated WBC	3.91 (0.93–16.44)	0.063
Elevated IgG	1.33 (0.32–5.51)	0.691
Elevated IgG1 level	1.24 (0.25–6.26)	0.794
Elevated IgG2 level	0.58 (0.14–2.30)	0.434
Elevated IgG3 level	1.94 (0.38–9.92)	0.428
IgA below LLN	5.79 (1.03–32.45)	0.046
Complement below LLN*	4.65 (0.47–46.28)	0.190

*LLN, lower limit of the normal range.

## Discussion

IgG4 related disease (IgG4-RD) is a fibroinflammatory disease with systemic involvement. The pathogenesis of IgG4-RD remains mostly unknown. Many studies have described blood and tissue eosinophilia in patients with IgG4-RD^5–11^. However, most of them didn’t describe the comparison between patients with and without eosinophilia. Only one study found that the presence of eosinophilia did not result in a different clinical pattern in patients with type 1 autoimmune pancreatitis^11^. However, in their analysis, they only included patients with single-organ involvement, not systemic IgG4-RD. Hence, the clinical significance of eosinophilia in IgG4-RD is still unclear.

In this study, based on the largest cohort of IgG4-RD patients in China, we report that 20% of patients had eosinophilia. Patients with and without eosinophilia showed different clinical patterns in terms of organ involvement and laboratory findings. Patients with eosinophilia seemed to have a higher disease burden as indicated by elevated serum IgG, IgG4, ESR, and more organs involved, and higher IgG4-RD RI score. As in an IgG4-RD grouping analysis which reported by Wallace *et al*.^17^, patients were divided into four groups, Group 4 was male-dominated, characterized by classic Mikulicz syndrome with systemic involvement, showing the highest serum IgG4 levels, but they did not give the comparison of blood eosinophil counts among groups. We previously revealed that systemic IgG4-RD patients with dacryoadenitis and sialadenitis (IgG4-DS) and internal organs involvement had higher serum IgG4 levels and a higher rate of eosinophilia than patients with only IgG4-DS^18^. Patients with only IgG4-DS are more likely to ignore the disease, thus delay the diagnosis and treatment until systemic symptoms occurred, this could be speculated by longer disease duration at diagnosis in patients with eosinophilia. When we independently analyzed the organ involvement number apart from lacrimal glands, salivary glands, and lymph nodes, there was no significant difference between the two groups.

Allergic disorders are common causes of reactive eosinophilia^19^. However, there was no statistically significant difference in the incidence of allergic disease between patients with and without eosinophilia, indicating that eosinophilia is independent of allergies in IgG4-RD patients, which was consistent with the results reported by Della-Torre *et al*.^10^ and Sah *et al*.^11^. Wallace *et al*.^20^ have found that elevated eosinophil count in the baseline was an independent predictor of disease relapse; also, our previous study showed eosinophilia was a risk factor for remission induction failure^21^. Here, we confirmed that IgG4-RD patients with peripheral eosinophilia were more liable to disease relapse.

We found here that peripheral eosinophil counts in IgG4-RD patients were positively correlated with serum IgG4 and disease burden. In addition, serum IgG4 level at 12 months was also positively correlated with the blood eosinophil count at 12 months. IgG4-RD exhibited activated Th2 response^22–26^, and this may be responsible for eosinophilia in IgG4-RD^19^. Otherwise, activated eosinophils release cytokines^2^, which can promote the production of IgG4^27^. Although it remains unclear that IgG4 plays a pathogenic or protective role in IgG4-RD^28^, these indicate that eosinophils participate in the pathogenesis of IgG4-RD.

Both CRP and ESR are used to predict inflammatory conditions. ESR level can be affected by factors other than inflammation, such as levels of serum fibrinogen and immunoglobulins. We believe that the difference of ESR levels between the two groups was mainly due to the different distribution of serum IgG and IgG4, as patients with eosinophilia had higher serum IgG, IgG4, while with a similar level of CRP compared to patients with normal eosinophil counts. Patients with eosinophilia showed male predominance, as reported that peripheral blood eosinophil count is skewed according to gender, males usually have higher eosinophil count^29,30^.

Activation of eosinophils led to the production of transforming growth factor (TGF)-β^2^, and the latter was reported to participate in the pathogenesis of fibrosis. Fibrosis occurred in the affected organs of advanced IgG4-RD patients. We found that peripheral eosinophil counts in IgG4-RD patients were positively correlated to treatment-free disease duration; patients with eosinophilia showed longer disease duration. These indicate that eosinophilia may occur in the advanced stage of IgG4-RD, and may participate in the fibrosis of affected tissues.

To date, several treatment approaches, including glucocorticoids, myelosuppressive drugs, leukotriene antagonists, tyrosine kinase inhibitors, IFN-α, and anti-IL-5 antibodies, have been described for eosinophilia. Although the role of eosinophilia in the pathogenesis of IgG4-RD is debatable, we found that IgG4-RD patients with peripheral eosinophilia tend to be more liable to disease relapse, this raises the possibility of anti-eosinophil therapy such as monoclonal antibody to IL-5 for IgG4-RD patients with recurrent disease.

Our study has some limitations. This is a single-center study, which may cause bias in interpreting results. Only two patients with eosinophilia had done bone marrow biopsy at baseline and showed elevated bone marrow eosinophils. No further tests for the myeloid or lymphocytic variant hypereosinophilic syndrome (HES) had performed. As proposed by Chen *et al*.^31^, the diagnosis of idiopathic HES need to rule out IgG4-RD, the differential diagnosis between IgG4-RD and myeloid or lymphocytic variant HES need careful consideration of clinical and laboratory manifestation.

In conclusion, peripheral eosinophilia was associated with a different clinical pattern of IgG4-RD. Eosinophil may play an important role in the development of IgG4-RD, especially in patients with longer disease duration and probably pronounced fibrotic lesion. Our findings paved the way for identifying the role of eosinophils in the pathogenesis of IgG4-RD.

## Methods

### Patients and study design

A multidisciplinary collaborative prospective cohort study of IgG4-RD and Mimicry patients has been conducted in Peking Union Medical College Hospital (PUMCH, Beijing, China) since January 2011, registered on ClinicalTrials.gov (ID: NCT01670695). Patients were diagnosed with IgG4-RD according to the 2011 comprehensive diagnostic criteria for IgG4-RD^32^. Fifty-one patients who had a history of other rheumatic diseases, infectious diseases, or malignancies, which may cause eosinophilia, were excluded. As of August 2018, 425 newly diagnosed IgG4-RD patients were enrolled in this study.

Eosinophilia in the peripheral blood was defined as eosinophil count greater than 500 per μL (0.5 × 10^9^ per L). We divided the patients into two groups according to whether there was eosinophilia. According to the Allergy Definition of the European Society for Allergy and Clinical Immunology^33^: (1) a history of clinical allergic diseases; (2) a positive skin test or an increase in serum allergen-specific IgE, while satisfying the definition of both as an allergy. Patients with newly diagnosed IgG4-RD were classified as allergic and non-allergic. All patients visited the doctors regularly at 1st and every 3 to 6 months intervals follow up.

Clinical data and laboratory parameters including complete blood count (CBC), liver and renal function tests, erythrocyte sedimentation rate (ESR), C-reactive protein (CRP), serum immunoglobulin levels, IgG subclasses, and total IgE levels were collected. Organ involvements were defined by physical examination and imaging findings, including computed tomography (CT), or magnetic resonance imaging (MRI), or Positron Emission Tomography/Computed Tomography (PET-CT). Disease activity was assessed by the IgG4-RD responder index (RI)^34^.

### Outcomes

Disease remission and relapse were described as before^21^. Briefly, the initial 6th -months of treatment was defined as the remission induction stage, and remission was defined as fulfilling each of the following after 6-month treatment: (1) ≥50% decline in the IgG4-RD RI; (2) GC tapered to maintenance dose (prednisone ≤10 mg/day); and (3) no relapse during GC tapering (within 6 months). Clinical symptoms recurred, or imaging findings worsened with or without IgG4 level increased were considered as disease relapse in patients who had a follow-up period of 3 months or longer.

### Statistical analysis

Continuous variables are described in mean and standard deviation (SD) or median and interquartile range (IQR) according to the data distribution. Comparisons between the two groups were performed using Student’s *t*-test or the appropriate non-parametrical test, depending on normal or non-normal data distribution. Categorical variables were expressed as percentages, and *χ*^2^-test was used for comparisons. Fisher’s exact test was used when one or more expected frequencies were less than 5. Correlation between two continuous non-normal variables was analyzed using Spearman’s rank correlation. Univariate logistic regression was used to predict risk factors for eosinophilia at 12 months follow-up. Statistical analysis was performed by SPSS version 24.0 and Graphpad Prism version 7.0. A *P*-value < 0.05 was considered statistically significant.

### Compliance with ethical standards

This study complied with the Declaration of Helsinki and was approved by the Human and Animal Ethics Review Committees of PUMCH, China (approval number: S-442). Informed consent was obtained from all individual participants included in the study.

## Data Availability

The datasets used and or analyzed during the current study are available from the corresponding author on reasonable request.
